# Podophyllotoxin and Rutin Modulates Ionizing Radiation-Induced Oxidative Stress and Apoptotic Cell Death in Mice Bone Marrow and Spleen

**DOI:** 10.3389/fimmu.2017.00183

**Published:** 2017-02-27

**Authors:** Abhinav Singh, M. H. Yashavarddhan, Bhargab Kalita, Rajiv Ranjan, Sania Bajaj, Hridayesh Prakash, Manju Lata Gupta

**Affiliations:** ^1^Division of Radioprotective Drug Development and Research, Institute of Nuclear Medicine and Allied Sciences, Defense Research and Development Organization, Delhi, India; ^2^Translational Medicine Laboratory, School of Life Sciences, University of Hyderabad, Hyderabad, India

**Keywords:** radioprotection, reactive oxygen species, oxidative stress, Nrf-2, antioxidant, apoptosis, p53, immunomodulation

## Abstract

The present study is aimed to investigate the radioprotective efficacy of G-003M (combination of podophyllotoxin and rutin) against gamma radiation-induced oxidative stress and subsequent cell death in mice bone marrow and spleen. Prophylactic administration of G-003M (−1 h) rendered more than 85% survival in mice exposed to 9 Gy (lethal dose) with dose reduction factor of 1.26. G-003M pretreated mice demonstrated significantly reduced level of reactive oxygen species, membrane lipid peroxidation, and retained glutathione level. In the same group, we obtained increased expression of master redox regulator, nuclear factor erythroid-derived like-2 factor (Nrf-2), and its downstream targets (heme oxygenase-1, Nqo-1, glutathione *S*-transferase, and thioredoxin reductase-1). In addition, G-003M preadministration has also shown a significant reduction in Keap-1 level (Nrf-2 inhibitor). Radiation-induced lethality was significantly amended in combination-treated (G-003M) mice as demonstrated by reduced 8-OHdG, annexin V FITC^+^ cells, and restored mitochondrial membrane potential. Expression of antiapoptotic protein Bcl-2 and Bcl-xL was restored in G-003M pretreated group. However, proapoptotic proteins (Puma, Bax, Bak, Caspase-3, and Caspase-7) were significantly declined in this group. Further analysis of immune cells revealed G-003M-mediated restoration of CD3 and CD19 receptor, which was found decreased to significant level following irradiation. Similarly, Gr-1, a marker of granulocytes, was also retained by G-003M administration prior to radiation. Modulatory potential of this formulation (G-003M) can be exploited as a safe and effective countermeasure against radiation-induced lymphohemopoietic injury.

## Introduction

Ionizing radiation (IR) manifests lymphohemopoietic injuries predominantly through increased generation of reactive oxygen species (ROS) (1). IR-induced cell death can be easily observed in bone marrow cells and splenocytes due to their high cell turnover rate (2). ROS is known to cause severe damage to cellular macromolecules such as DNA, proteins, and lipids (3, 4). Damage to DNA leads to apoptotic cell death, necrosis, and inflammation (5, 6). Immune cells are known to be highly vulnerable to radiation, through induced apoptosis in mature T and B lymphocytes and by lethal damage in bone marrow stem cell precursors of monocytes and granulocytes. Apart from targeted effect, indirect effect of IR, such as bystander effect and inflammation have also been demonstrated to cause severe lethality (7). Exposure to high radiation dose can cause severe reduction in the hemopietic stem and progenitor cell of bone marrow and lymphocytes of spleen, which may cause immunosuppression (8) and subsequently, leads to various malaise, opportunistic infection, and mortality in exposed organism. Therefore, protection to the hematopoietic and lymphoid system is extremely important to mitigate IR-induced lethality.

Ionizing radiation activates both pro- and antiproliferative signal pathway by altering the homeostatic balance between cell survival and cell death. This phenomenon is regulated by several transcriptional factors and genes involved in DNA damage and repair, cell cycle arrest, cell death antioxidation, and inflammation. Cellular machinery has the capacity of lowering the ROS levels produced following irradiation by their antioxidant machinery. However, excessive production of ROS during exposure to large doses of radiation jeopardizes the antioxidant machinery and causes a pathological state termed as necroptosis oxidative stress (9). The cellular system attempts to ameliorate oxidative stress by activation of master redox regulator and an important pro-survival transcriptional factor, nuclear factor erythroid-derived like-2 factor (Nrf-2) (10). Nrf-2 augments gene expression of various antioxidants, detoxifying and cytoprotective proteins [heme oxygenase-1 (Ho-1), NAD(P)H:Quinone Oxidoreductase 1 (Nqo-1), thioredoxin reductase-1 (Txnrd-1), and glutathione *S*-transferase (Gst)] (11). Exorbitant level of ROS also induces permeabilization of the mitochondrial membrane that releases various proapoptotic stimuli from mitochondria to cytosol, resulting in the activation of various proapoptotic genes that ultimately leads to apoptotic cell death. Transcriptional factor, p53, mediates transactivation of various proapoptotic proteins involved in the induction of apoptotic cell death (12). However, antiapoptotic proteins Bcl-2 and Bcl-xl are another important regulators of cell death pathway, which have been demonstrated for its inhibitory effect on various proapoptotic proteins.

Prophylactic administration of the molecules having strong antioxidant, anti-inflammatory, and immunomodulatory property are considered to be the prime approach for development of the radioprotectors. Several compounds of synthetic origin, such as amino thiols (Amifostine), nitroxides (Tempol), and DNA-binding agents (Hoechst 33342), have shown significant increment in post-irradiation mice survival (13, 14). In addition, tocopherol succinate, 4-carboxystyryl-4-chlorobenzyl sulfone sodium salt (0N01210.Na/Ex-Rad), Simvastatin, CBLB613, KR22332, and histamine derivatives have also been demonstrated for their radiomodulatory potential (15–17). Some of these compounds have advanced up to various clinical trial phases, but failed due to many reasons (18). Amifostine, however, is the only chemical compound that is clinically approved by the United States Food and Drug Administration (19) for limited clinical application.

In addition, large numbers of natural resources (plants, minerals, vitamin, and antioxidants) have also been examined for their radioprotective ability in the recent past. Herbs are amply known to be rich in antioxidants, immunostimulant, anti-inflammatory, and antimicrobial agents having minimal or negligible toxicity. These multifaceted properties and negligible/minimal toxicity made phytocompounds more advantageous over synthetic compound (20). Out of the various herbs, *Podophyllum hexandrum* has been extensively explored by our group for its radioprotective efficacy. Our previous studies have demonstrated the radioprotective effect of *P. hexandrum* in various *in vitro, ex vivo*, and *in vivo* model systems against sublethal and lethal radiation exposures. This plant is profoundly rich in a number of bioactive constituents, mainly lignans, flavonoids, and their glucosides (21). Our earlier formulation (combination of podophyllotoxin, β-d-glucoside, and rutin) has already been reported for more than 85% survival in lethally irradiated mice. This has happened predominantly by formulation-mediated efficient scavenging ROS (22), upregulation of DNA repair proteins (23), reduced inflammation (i-nos formation) (24), etc.

However, the current study is designed to investigate the protective efficacy of G-003M (combination of podophyllotoxin and rutin) against lethal radiation-induced damage to mice bone marrow and spleen. Podophyllotoxin has been demonstrated for its DNA-protecting ability by reversible cell cycle arrest (G2/M) *via* inhibition of tubulin polymerization (25, 26). During this stage, cells remain in quiescent stage and therefore are more radioresistant. As a result, minimal damage occurs to DNA and haulted cell cycle further provides enough time for cells to undergo DNA repair (27). Rutin, the other component of G-003M is a well-known antioxidant and anti-inflammatory compound (28, 29). Both the compounds, i.e., podophyllotoxin and rutin, alone as well as in combination (G-003M) have also been demonstrated for their radiomodulatory efficacy while estimating expression of Nrf-2, p53, and Gr-1. Some parameters of current study have also been performed with the use of amifostine as a positive control.

Present study demonstrates G-003M-mediated regulation of IR-induced ROS formation, membrane lipid peroxidation, non-protein thiol glutathione (GSH) depletion, mitochondrial membrane potential (MMP) alteration, and oxidative damage to DNA (8-OH-dG). G-003M preadministered mice has shown significantly regulated level of various proapoptotic (p53, Puma, Bax, Bak, Caspase-3, and Caspase-7) and antiapoptotic proteins (Bcl-2 and Bcl-xl). Further analysis revealed G-003M-mediated induction in the master redox regulator, Nrf-2, and its several downstream target proteins (Nqo-1, Ho-1, Gst, and Txnrd-1) through negative regulation of Keap-1. Mice pretreated with G-003M had also shown significant recovery to CD3, CD19, and Gr-1 cell surface marker in mice bone marrow and spleen, which otherwise was significantly declined following irradiation.

## Materials and Methods

### Reagents

Acrylamide, bis-acrylamide, trizma base, sodium dodecyl sulfate, glycine, ammonium per sulfate, TEMED, KCL, Na_2_HPO_4_, K_2_HPO_4_, NH_4_CL, K_2_HCO_3_, EDTA, BSA, tween-20, triton-X-100, paraformaldehyde, methanol, DMSO, acetic acid, HCL, bradford, cocktail of protease inhibitors, gel loading buffer, mito-red, DCF-DA, and ECL chemiluminescent kit were procured from the Sigma-Aldrich (St. Louis, MO, USA). Primary antibodies like anti Nrf-2 (Cat no. ab31163), anti Ho-1 (Cat no. ab13248), anti Nqo1 (Cat no. ab28947), anti-keap-1 (Cat no. ab150654), anti-Gst (Cat no. Ab 53940), anti-Txnrd-1 (Cat no. Ab124954) anti p53 (Cat no. ab26), anti-Puma (Cat no. ab9643), anti Bax (Cat no. ab5714), anti Bak (Cat no. ab104124), anti-caspase-3 (Cat no. ab44976), anti-caspase-7 (Cat no. ab69540), anti Bcl-2 (Cat no. ab692), anti Bcl-xl (Cat no. ab32370), and 8-OH-dG (Cat no. ab201734) were obtained from the Abcam (Cambridge, MA, USA). Anti-CD3-PE conjugated, anti-CD19-FITC conjugated, and anti-Ly6g (Gr-1)-PE conjugated antibodies were procured from BD Biosciences (San Jose, CA, USA). Annexin V FITC apoptosis detection kit (Cat no. PF032) was purchased from Calbiochem. Anti β-actin (Cat no. 04-1116), secondary antibody goat anti-rabbit IgG (H + L) FITC conjugate (Cat no. AP307F), goat anti-rabbit IgG (H + L) HRP conjugate (Cat no. AP307P), goat anti-mouse IgG (H + L) FITC conjugate (Cat no. AP308F), and goat anti-mouse IgG (H + L) HRP conjugate (Cat no. AP308P) were procured from the Millipore (CA, USA).

### Preparation of G-003M Formulation

G-003M is the combination of two phytocompounds, podophyllotoxin and rutin. The effective formulation was prepared initially by mixing both the compounds in different permutation and combinations. The ratio we used in the current study was 1:2 of compound A (podophyllotoxin) and B (Rutin). G-003M was prepared fresh at the time of administration by dissolving both the compounds in DMSO. The solution was further diluted in distilled water to a final ratio of 1:9 (DMSO:water). The 10% DMSO was used for the formulation preparation. The preparation was administered intramuscularly (150 µl per mice at a dose of 6.5 mg/kg body weight) 1 h prior to radiation exposure. The effective concentration of the formulation was obtained from the whole-body survival study as an end point. However, the most effective time point for formulation administration was obtained from time window study of G-003M.

For positive control, amifostine was used at a concentration of 100 mg/kg body weight. Amifostine was freshly dissolved in sterile distilled water and was subcutaneously administered to mice 30 min prior to radiation exposure.

### Animal Studies

The study design strictly adhered to the guidelines approved by Institutional Animal Care and Use Committee (IACUC) of our institute, Institute of Nuclear Medicine and Allied Sciences (INMAS) (INM/IAEC/2013/03, dated 06.06.2013). Mice obtained from the Institutional Animal Facility, were maintained at 20–22°C and relative humidity of 50–70%. Mice were given a standard diet of rodent pellets (Golden feed Pvt. Ltd., Delhi, India) and water *ad libitum*. Six to eight week old strain “A” female mice were restrained in well-ventilated perplex boxes and exposed to whole-body gamma irradiation (9 Gy) in the ^60^Cobalt gamma chamber (Cobalt Teletherapy, Bhabhatron II, Panacea Medical Technologies Pvt. Ltd., India) at a dose rate of 0.9864 Gy/min. The exposure window and source to surface distance was 35 × 35 and 120 cm, respectively. Immediately after irradiation, mice were returned to the cage and rested. Radiation dose calibration was performed by the institutional radiation physicists at regular time intervals by Frick’s dosimetry.

For survival assay, mice were randomly divided into four groups (vehicle (DMSO) + 9 Gy, 9 Gy, G-003M + 9 Gy, and G-003M only) of six mice in each group and experiment was repeated thrice. Data obtained from three experiment (*N* = 18 mice/experimental group) were statistically analyzed and represented by a Kaplan–Meier survival curve. For time response study of various proteins, mice (*N* = 6) were sacrificed at various time intervals following irradiation (6, 12, and 24 h). For observing the modulatory effect of G-003M, mice were randomly divided into four groups (control, G-003M alone, 9 Gy, G-003M + 9 Gy) of six mice in each group and sacrificed majorly at 24 h (48 and 72 h in some parameters) post-exposure and each experiment was repeated twice. Figure S4D in Supplementary Material depicts the number of animals used in different experimental groups. For estimation of dose reduction factor (DRF), irradiated mice with or without pretreatment with G-003M (6–12 Gy) were observed for a period of 30 days to calculate the LD50/30. In the time window study, G-003M was administered at various time intervals (−240 to +30 min pre- or post-irradiation) and survival percentage was recorded as a function of time. The most effective time interval (−1 h) was used for G-003M administration during the entire study.

### Primary Cell Suspension

Mice were sacrificed by cervical dislocation and spleen and femur was aseptically isolated and placed in sterile micro centrifuge tubes containing ice-cold phosphate-buffered saline (PBS). Single-cell suspension was prepared by gently mincing the spleen between the two frosted glass slides by continuous pouring with PBS. Bone marrow cells were isolated by flushing the femur bone with the 24 gauge needle using PBS. Bone marrow and splenocytes were then centrifuged at 1,000 *g* for 8 min at RT. RBCs were lysed by potassium bicarbonate buffer. After RBC lysis, cells were washed with the ice-cold PBS twice. Cell viability was determined by trypan blue dye exclusion assay, and purified bone marrow cells and splenocytes were directly used for various cellular assays.

### Lipid Peroxidation

Briefly, spleen isolated at 24 and 48 h post-exposure was homogenized in lysis buffer (50 mM Tris–Cl, 1% NP-40, 0.2% sodium deoxycholate, 0.1% SDS, 150 mM NaCl, and 1 mM EDTA). Lipid peroxidation was performed as per the method of Buege and Aust (30), by measuring the levels of thiobarbituric acid reactive species (TBARS), using malonaldehyde (MDA) as a standard. Spleen cell lysate (1 ml) was mixed with the 2 ml of TCA (15% w/v), TBA (0.375% w/v), and 0.25 N HCL, followed by incubation at 90°C for 30 min. After cooling, the reaction mixture was centrifuged at 10,000 *g* to remove the precipitate. Absorbance of supernatant was taken at 535 nm against the blank. Amount of lipid peroxidation was expressed in terms of TBARS in nanomoles per milligram of protein, which was estimated by using a value of ε = 1.56 × 10/M/cm.

### GSH Estimation

Spleen tissue, obtained at 24 and 48 h post-experimentation, was homogenized in lysis buffer (50 mM Tris–Cl, 1% NP-40, 0.2% sodium deoxycholate, 0.1% SDS, 150 mM NaCl, and 1 mM EDTA). GSH was assessed using the method of Ellman (31). Assay mixture consisted of 0.2 ml of tissue homogenate, 1.8 ml of (0.5 M) EDTA solution, and 3.0 ml precipitating reagent (In 1 l–1.67 g of meta-phosphoric acid, 0.2 g of EDTA disodium salt, and 30 g sodium chloride). After mixing thoroughly, solution was kept for 5 min and then centrifuged. This step helps in separating the GSH (in the supernatant) from the rest of the protein and other cellular entities (in precipitate). A total of 4.0 ml (0.3 M) disodium hydrogen phosphate solution and 1.0 ml of DTNB (5, 5-dithio-bis-2-nitrobenzoic acid) was then added to 2.0 ml of the supernatant. Absorbance was taken at 412 nm against blank. GSH was measured in nanomol/mg protein using a standard curve.

### ROS Measurement

1 × 10^6^ viable bone marrow cells and splenocytes obtained at 1 h post-exposure from differently treated mice were washed with the PBS and incubated with the oxidation sensitive dye di-chlorofluorescein diacetate (DCF-DA, 10 µM) for 30 min in dark at 37°C. After incubation, cells were washed with the PBS and change in fluorescence resulting from oxidation of H2DCF to DCF was measured by the flow cytometry (BD Biosciences, San Jose, CA, USA).

### Mitochondrial Membrane Potential

For estimation of MMP, 1 × 10^6^ viable bone marrow cells and splenocytes isolated at 24 h post-experimentation were washed with the PBS and incubated with the 40 nM mito-red in dark at 37°C. After incubation, cells were washed with the phosphate buffer and analyzed by flow cytometry (BD Biosciences, San Jose, CA, USA).

### Measurement of 8-Hydroxy-2-Deoxyguanosine (8-OH-dG)

8-OH-dG was measured in mice plasma using a commercial 8-OH-dG ELISA kit (Abcam) following the manufacturer’s protocol. Plasma was diluted at a ratio of 1:20 in sample and standard dilutant and concentration (nanograms per milliliter) was measured at 450 nm.

### Cell Death Analysis

Annexin V FITC/PI assay was used to quantify the percentage of apoptotic cells in bone marrow cells and splenocytes at 24 h post-irradiation. 1 × 10^6^ live cells were stained with annexin V FITC/PI (Apoptosis detection kit Millipore) following the manufacturer’s protocol. For the assay, cells were washed with the PBS and incubated in 1× binding buffer (10 mM HEPES/NaOH, pH 7.4, 140 mM NaCl, and 2.5 mM CaCl_2_). Afterward, cells were incubated with annexin V FITC and PI for 15 min at room temperature in dark. Cells were then acquired and analyzed by flow cytometry (FACS Calibur, Becton Dickinson, USA).

### Immunoblotting

Bone marrow and splenocytes were washed with the PBS and lysed in radio immune precipitation buffer (50 mM Trizma base, pH 8.0, 150 mM NaCl, 0.2 mM EDTA, 1% NP-40, 0.5% sodium deoxycholate, 1 mM PMSF, and 1 mM sodium orthovandate supplemented with 1× protease inhibitor mixture) for 30 min at 4°C. After lysis, cells were centrifuged at 8000 *g* for 15 min at 4°C. Protein concentration was determined by Bradford assay. Thirty micrograms of proteins were resolved on SDS-PAGE and transferred onto nitrocellulose membrane. After transfer of the proteins, membrane was blocked with the 5% non-fat dried milk in PBS with 0.1% Tween-20 for 2 h at room temperature. Membrane was then incubated with the primary antibody overnight at 4°C under shaking condition. After washing with the PBST, membrane was incubated with either goat anti-mouse or goat anti-rabbit IgG-HRP (at 4°C for 3 h) and blots were then visualized using an enhanced chemiluminescent kit (Sigma-Aldrich, St. Louis, MO, USA). Intensity of protein bands was quantified using image lab software (Gel Doc XR+, Biorad).

### Flow Cytometry

Bone marrow cells and splenocytes isolated from differently treated mice were fixed in 4% paraformaldehyde for 30 min at 4°C for intracellular staining. After fixation, cells were washed with PBS and treated with 0.5% glycine for 15 min to quench the remaining paraformaldehyde from cells. Cells were permeabilized with 0.1% Triton-X-100 for 15 min at RT and blocked with 2% bovine serum albumin for 1 h at RT. After blocking, cells were stained with the primary antibodies overnight at 4°C at shaker. Cells were then washed with the PBS and incubated with the fluorophore tagged respective secondary antibodies for 2 h at 4°C. After washing with phosphate buffer, cells were acquired by FACS caliber and data were analyzed by CellQuest software (BD Biosciences).

For surface staining/direct staining of CD3, CD19, and Gr-1, 1 × 10^6^ viable splenocytes and bone marrow cells were washed with the ice-cold phosphate buffer and fixed in70% ethanol. Cells were then blocked with bovine serum albumin (1%) for 30 min. After blocking, cells were stained with PE-conjugated anti-CD3, FITC-conjugated anti-CD19, and PE-conjugated anti-Gr-1 antibodies for 2 h at RT. After staining, cells were again washed with the ice-cold PBS, acquired, and analyzed by FACS Caliber. Data were analyzed by CellQuest software (BD Biosciences).

### Statistical Analysis

The data obtained are represented as mean ± SEM. The difference between the experimental groups was evaluated by one-way analysis of variance, with Newman–Keuls multiple comparison test (V, 5.01; GraphPad Prism, San Diego, CA, USA). Assumption used for hypothesis testing and measured quantity is not used in the current study. For animal survival assay, Kaplan–Meier analysis was used. A value of *P* less than 0.05 was considered statistically significant.

## Results

### G-003M Extended the Survival of Lethally Exposed Mice

**Figure 1:**
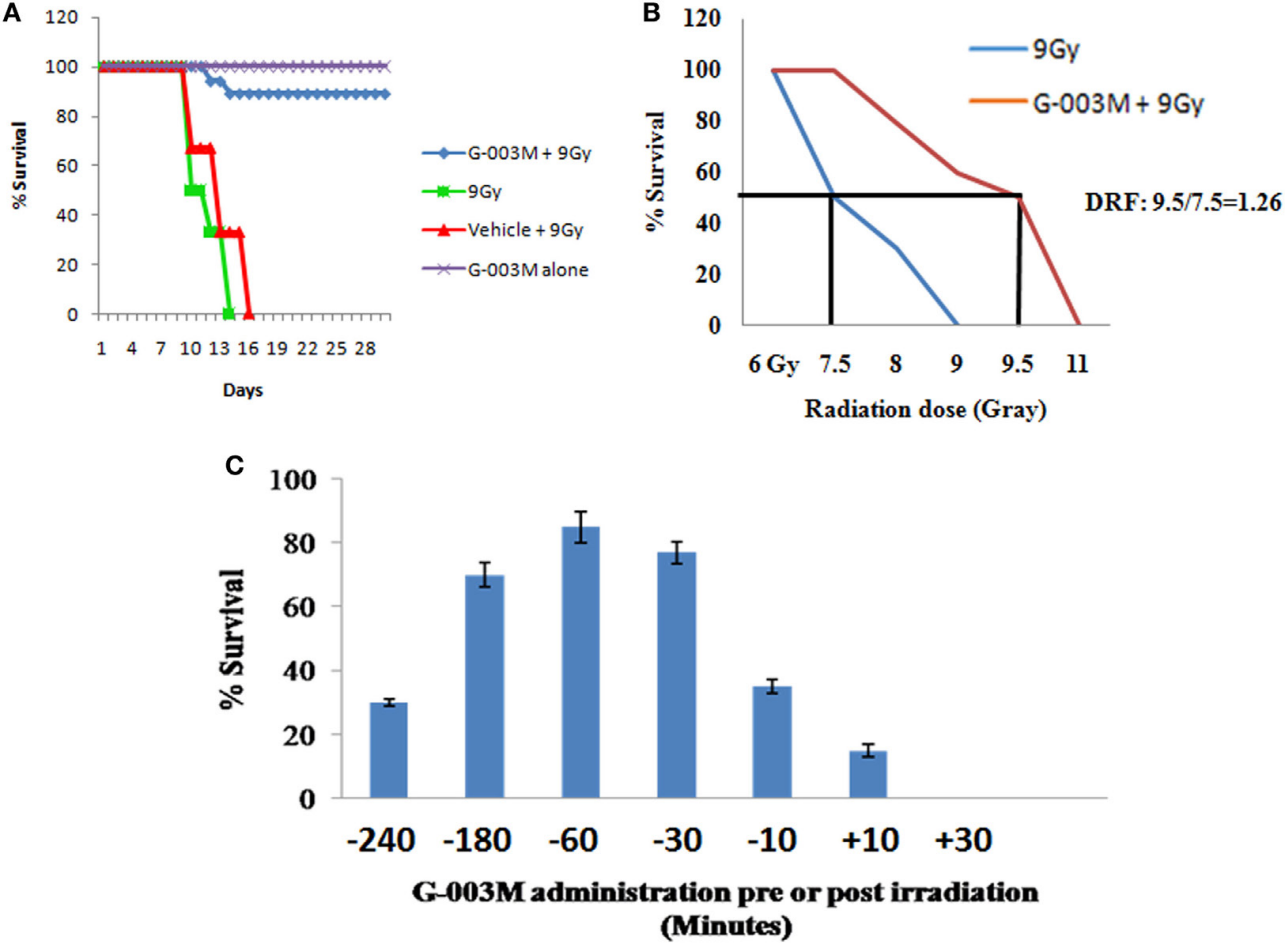
**Effect of G-003M on survival of mice exposed to radiation**. **(A)** G-003M extended the survival of whole-body irradiated mice. Mice were intramuscularly preadministered (−1 h) with a single and therapeutically relevant dose of G-003M (6.5 mg/kg body weight) and exposed to whole-body irradiation (9 Gy). For survival assay, mice were randomly divided into four groups (vehicle (DMSO) + 9 Gy, 9 Gy, G-003M + 9 Gy, and G-003M only) of six mice in each group and observed for a period of 30 days for radiation-induced morbidity and mortality. Survival experiment was repeated thrice. Data obtained (*N* = 18) was statistically analyzed and represented by a Kaplan–Meier survival curve. **(B)** For estimation of dose reduction factor (DRF), mice were treated with G-003M and then irradiated. A ratio of LD50/30 dose of radiation with or without G-003M pretreatment has revealed a DRF of 1.26. **(C)** Estimation of most effective time interval for drug administration (pre- or post-irradiation).

Radioprotective efficacy of G-003M was assessed in strain “A” mice (Figure 1). Our finding revealed a gradual decrease in body weight, intake of food, water, and 100% mortality within 15 days post-exposure in irradiated group. However, we observed more than 85% protection and rescue in body weight loss by G-003M against 0% survival in radiation-exposed group (Figure 1A). The DRF reflecting the protective efficacy of the molecules against IR-induced lethality was 1.26 in case of our formulation. G-003M preadministration to mice could shift LD50/30 from 7.5 Gy (radiation-only group) to 9.5 Gy in formulation pretreated and irradiated group (Figure 1B). The G-003M administration at different time intervals (30 min to 3 h) prior to irradiation did not reveal any significant change in survival index. However, the survival efficacy was observed to be decreased when formulation was administered less than 30 min and more than 3 h prior to radiation exposure. G-003M administration leads to approximately 18% survival at 10 min and 0% survival at 30 min post-exposure. Based on the observation, −1 h was selected as the optimum time for drug administration (Figure 1C). Therapeutically optimum dose of G-003M was obtained by permutation and combination of its constituents and their effect on survival of the animal.

### G-003M Attenuated Markers of IR-Induced Oxidative Stress

**Figure 2:**
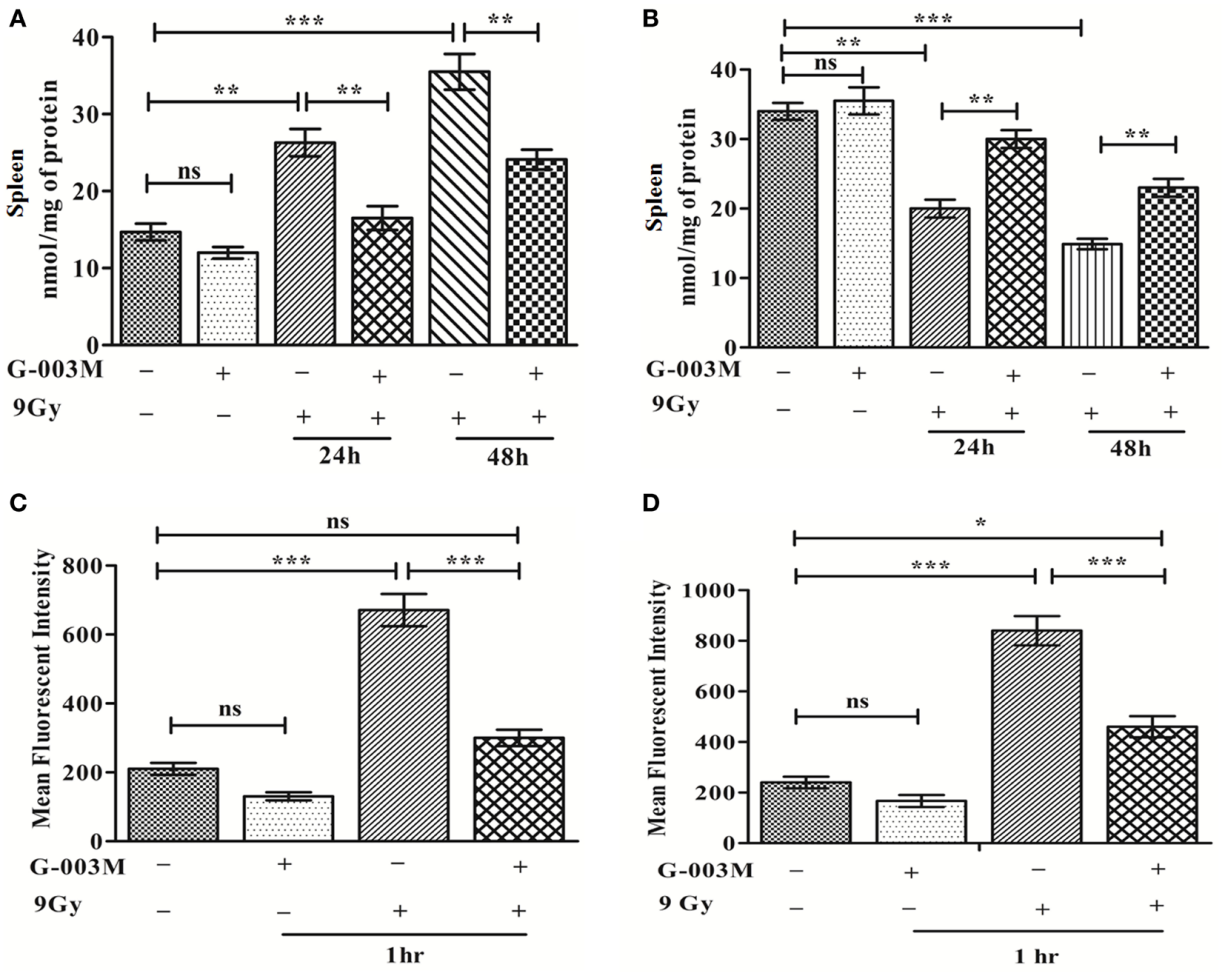
**G-003M attenuates ionizing radiation-induced membrane lipid peroxidation, glutathione (GSH) depletion by regulating reactive oxygen species (ROS) level**. **(A)** MDA level in spleen. **(B)** GSH level in spleen. **(C)** Intracellular production of ROS in bone marrow. **(D)** Level of ROS generation in splenocytes. Data represent mean ± SEM of six mice and experiment was repeated twice. The statistical differences between different experimental groups were compared. A value of *P* ≤ 0.5 is considered statistically significant (ns, non-significant, *P ≤ 0.05, **P ≤ 0.01, and ***P ≤ 0.001).

Any pharmacological agent, having the potential of mitigating radiation injury, should potentially modulate ROS and ROS-induced oxidative stress. As expected, pretreatment of G-003M significantly reduced IR-induced level of MDA in mice spleen at 24 and 48 h post-exposure. G-003M-alone treated group revealed non-significant change in MDA level when compared to untreated group (Figure 2A). Accordingly, G-003M pretreatment significantly enhanced GSH levels in mice spleen, which was reduced drastically following radiation exposure, suggesting the antioxidant role of this formulation. GSH level in G-003M-alone treated mice group was comparable to sham group (Figure 2B). In line with the above findings, the G-003M preadministration also markedly reduced radiation-mediated intracellular generation of ROS (*P* ≤ 0.001) in both the bone marrow (Figure 2C) and splenocytes at 1 h post-exposure (Figure 2D). Flow cytometric histogram representing the level of ROS in mice bone marrow and spleen is demonstrated in Figures S1A,B in Supplementary Material, respectively.

### G-003M Regulates MMP, Oxidative DNA Damage, and Subsequent Apoptosis

**Figure 3:**
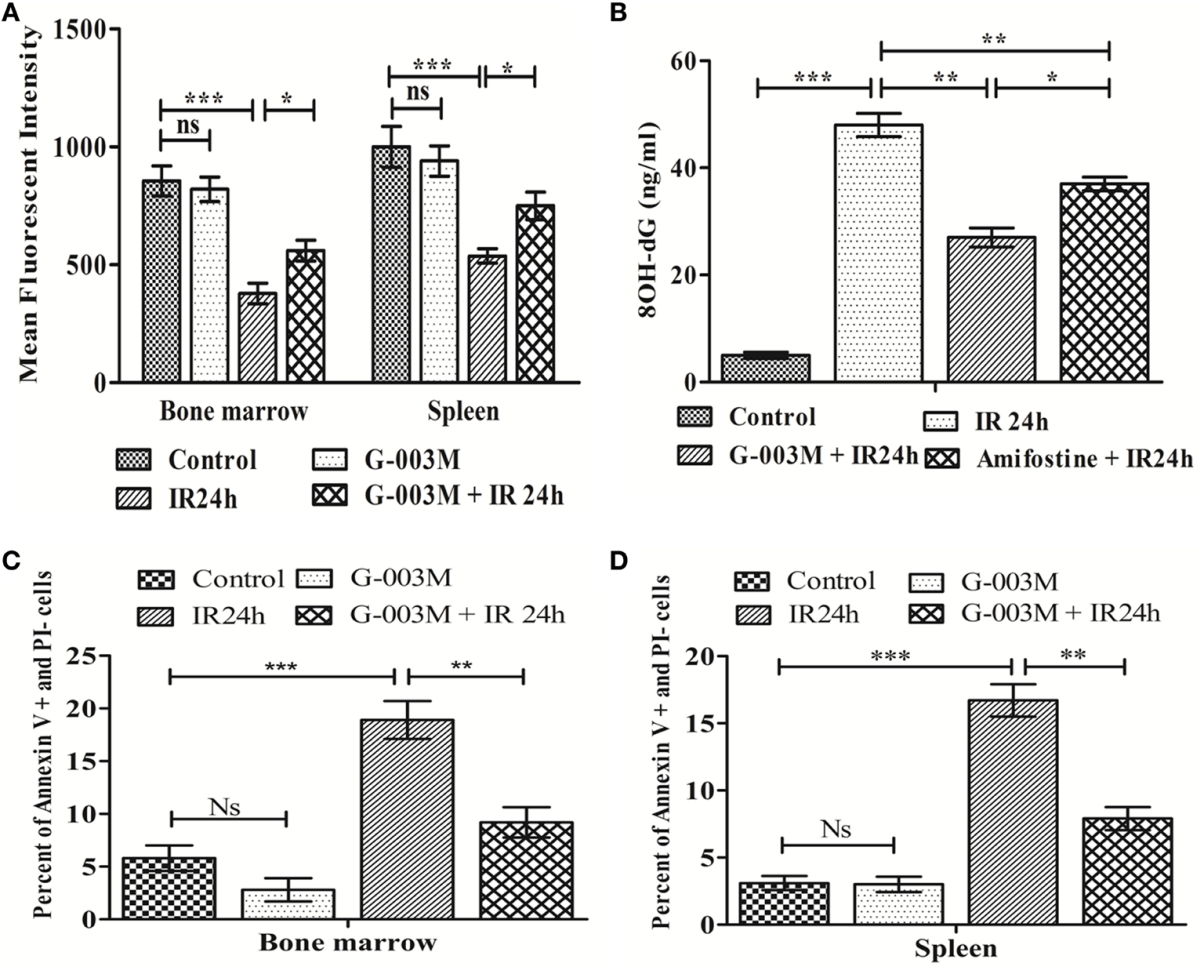
**Regulation of ionizing radiation (IR)-induced alteration in mitochondrial membrane potential, oxidative damage to DNA, and apoptotic cell death by prophylactic administration of G-003M**. **(A)** Bar diagram representing the mean fluorescence intensity of mito-red in bone marrow and splenocytes. **(B)** Bar diagram showing plasma level of 8-OH-dG. **(C)** Data depict percent apoptotic cell in bone marrow. **(D)** Data showing percentage apoptotic cells in splenocytes. Data represent mean ± SEM of six mice and experiment was repeated twice. The statistical differences between different experimental groups were compared. A value of *P* ≤ 0.5 is considered statistically significant (ns, non-significant, *P ≤ 0.05, **P ≤ 0.01, and ***P ≤ 0.001).

Disturbance in MMP is considered as a prerequisite for IR-induced apoptosis. IR significantly reduced MMP level in bone marrow and splenocytes (Figure 3A) at 24 h following irradiation when compared to controls (*P* ≤ 0.001). G-003M treatment to mice prior to irradiation, however, significantly retained the MMP level (*P* ≤ 0.01) in both the bone marrow and spleen. A flow cytometric histogram of MMP in both the organs is depicted in Figures S1C,D in Supplementary Material.

8-OH-dG is a marker of oxidative damage to DNA. Following irradiation (24 h), plasma level of 8-OH-dG was found significantly increased when compared with the untreated group (*P* ≤ 0.001). However, pre-irradiation administration of G-003M had markedly reduced 8-OH-dG in comparison to radiation alone group (*P* ≤ 0.01). On replacement of G-003M with the amifostine though the level of 8-OH-dG decreased, however, value of 8-OH-dG was still high in amifostine treated group when compared with G-003M treated group (Figure 3B).

Ionizing radiation also led to a significant increase in percent apoptosis (Annexin V FITC^+^ and PI^−^) in bone marrow cells at 24 h post-exposure (Figure 3C). As expected, priming of mice with G-003M protected hemopoietic stem cells of bone marrow from radiation-induced lethality. More or less similar trend was also obtained in splenocytes (Figure 3D). No significant change in percent of annexin V FITC^+^/PI^−^ cells was observed in these cells when G-003M-only administered mice was compared with untreated mice, suggesting the non-toxic nature of this formulation. A flow cytometric dot plot of this measurement has been demonstrated in the Figures 2A,B in Supplementary Material.

**Figure 4:**
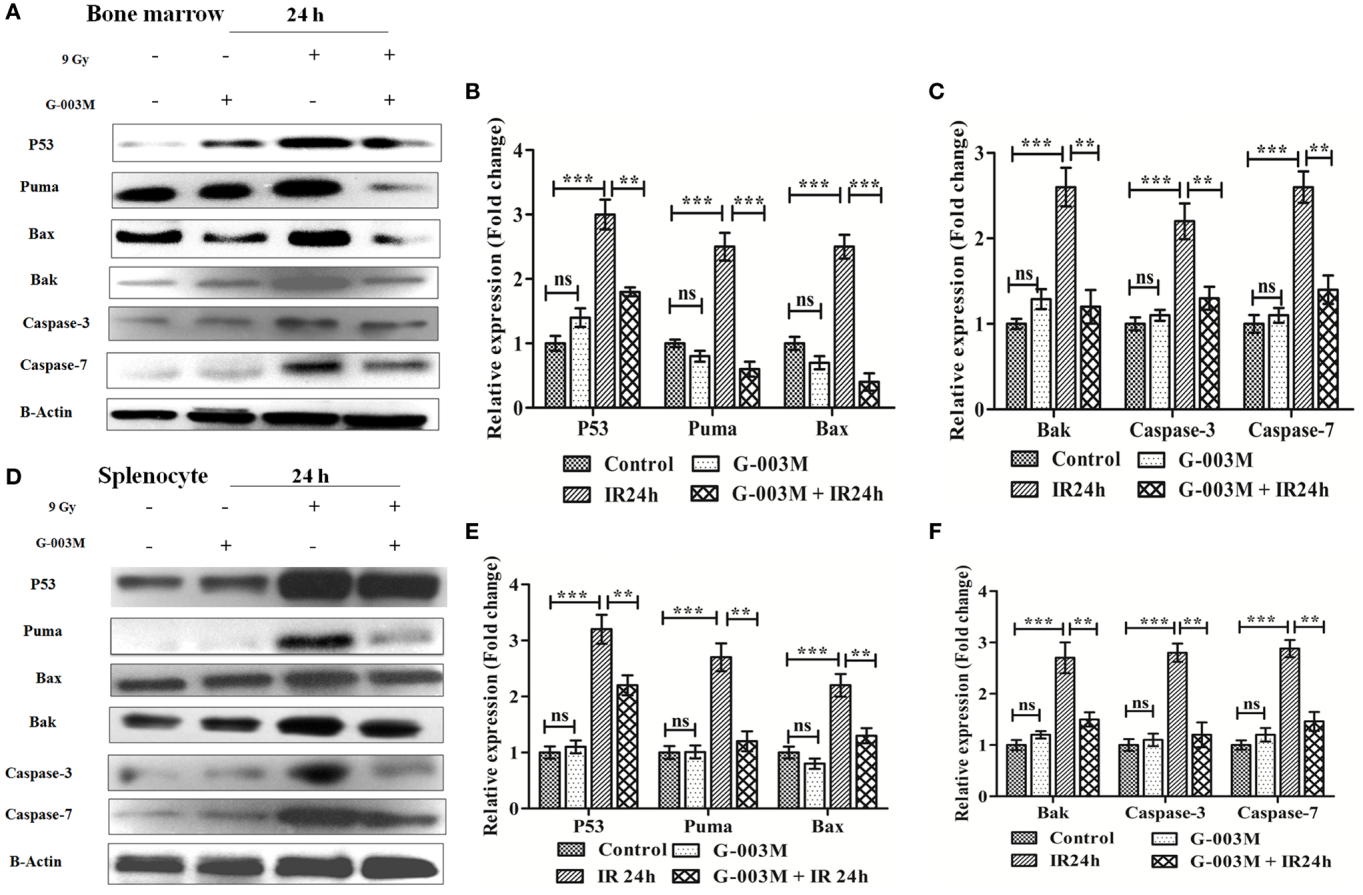
**G-003M intervention modulates expression of various proapoptotic proteins in radiation exposed mice**. **(A)** Immunoblot of proapoptotic proteins in bone marrow cells. **(B,C)** Bar diagram depicting the fold change in expression of proapoptotic proteins in bone marrow. **(D)** Immunoblot of apoptotic markers in splenocytes. **(E,F)** Bar diagram demonstrating fold change in expression of proapoptotic proteins in splenocytes. Data represent mean ± SEM of six mice and experiment was repeated twice. Statistical differences in various experimental groups were compared. A value of *P* ≤ 0.5 is considered statistically significant (ns, non-significant, **P ≤ 0.01, and ***P ≤ 0.001).

To further elucidate the radiomodulatory effect of G-003M, we have measured expression levels of various proapoptotic proteins in bone marrow and splenocytes through immunoblotting. Time response study of apoptotic proteins in both the bone marrow and spleen revealed a time-dependent expression (Figure S3 in Supplementary Material). Expression levels of various proapoptotic proteins (p53, puma, bax, bak, caspase-3, and caspase-7) was significantly (≥2-fold) increased at 24 h post-exposure when compared with controls. However, G-003M pretreatment to mice significantly reduced expression of these proteins in both the bone marrow (Figures 4A–C) and spleen (Figures 4D–F). Mice treated with G-003M alone also demonstrated reduced expression of these proteins in contrast to untreated mice.

**Figure 5:**
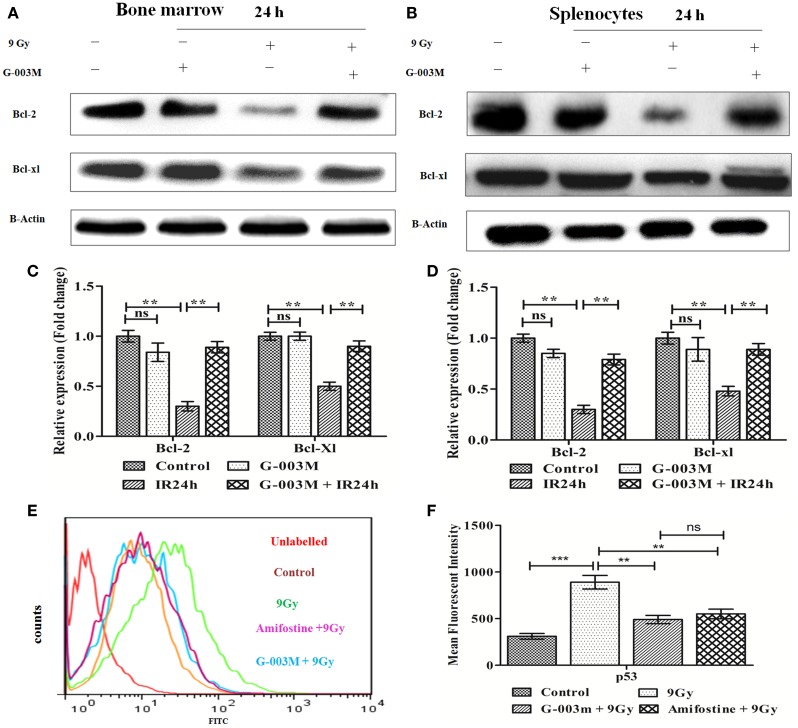
**G-003M pretreatment restored the expression of antiapoptotic proteins Bcl-2 and Bcl-xl in irradiated mice**. **(A)** Immunoblot of Bcl-2 and Bcl-xl in bone marrow. **(B)** Immunoblot of Bcl-2 and Bcl-xl in splenocyte. **(C)** Bar diagram depicting fold change in expression of proteins in bone marrow cells. **(D)** Data showing fold change in expression of proteins in splenocyte. **(E)** Flow cytometric overlaid histogram of p53 in bone marrow with use of amifostine as a positive control. **(F)** Bar diagram showing mean fluorescent intensity of p53. Data represent mean ± SEM of six mice and experiment was repeated twice. Statistical differences between various experimental groups were compared. A value of *P* ≤ 0.5 is considered statistically significant (ns, non-significant, **P ≤ 0.01, and ***P ≤ 0.001).

To explore the antiapoptotic property of G-003M, expression of Bcl-2 and Bcl-xl was also estimated in bone marrow cells and splenocytes at 24 h post-exposure by immunoblotting. Radiation exposure led to significant down-regulation of Bcl-2 and Bcl-xl in bone marrow cells when compared to control (*P* ≤ 0.01) (Figures 5A,C). G-003M pretreated and irradiated group, however, demonstrated significantly retained level of both the proteins in contrast to radiation alone group (*P* ≤ 0.01). Similarly, in spleen also, G-003M preadministration could significantly restore the level of Bcl-2 and Bcl-xl (*P* ≤ 0.01) (Figures 5B,D).

The expression of p53 was also evaluated using amifostine in bone marrow. Observation of this study revealed significantly reduced level of p53 in G-003M and amifostine pretreated mice when compared with radiation-exposed mice. However, p53 level was more or less similar in both the groups (G-003M and amifostine) (Figures 5E,F). In addition, a direct effect of both the compounds, i.e., podophyllotoxin, rutin, and their combination on p53 expression have also been assessed in bone marrow cells. Combination treated mice showed significantly reduced p53 in comparison to either podophyllotoxin or rutin alone treated mice (Figure S4A in Supplementary Material).

### Modification of Oxidative Stress/Nrf-2 Signaling by G-003M Intervention

**Figure 6:**
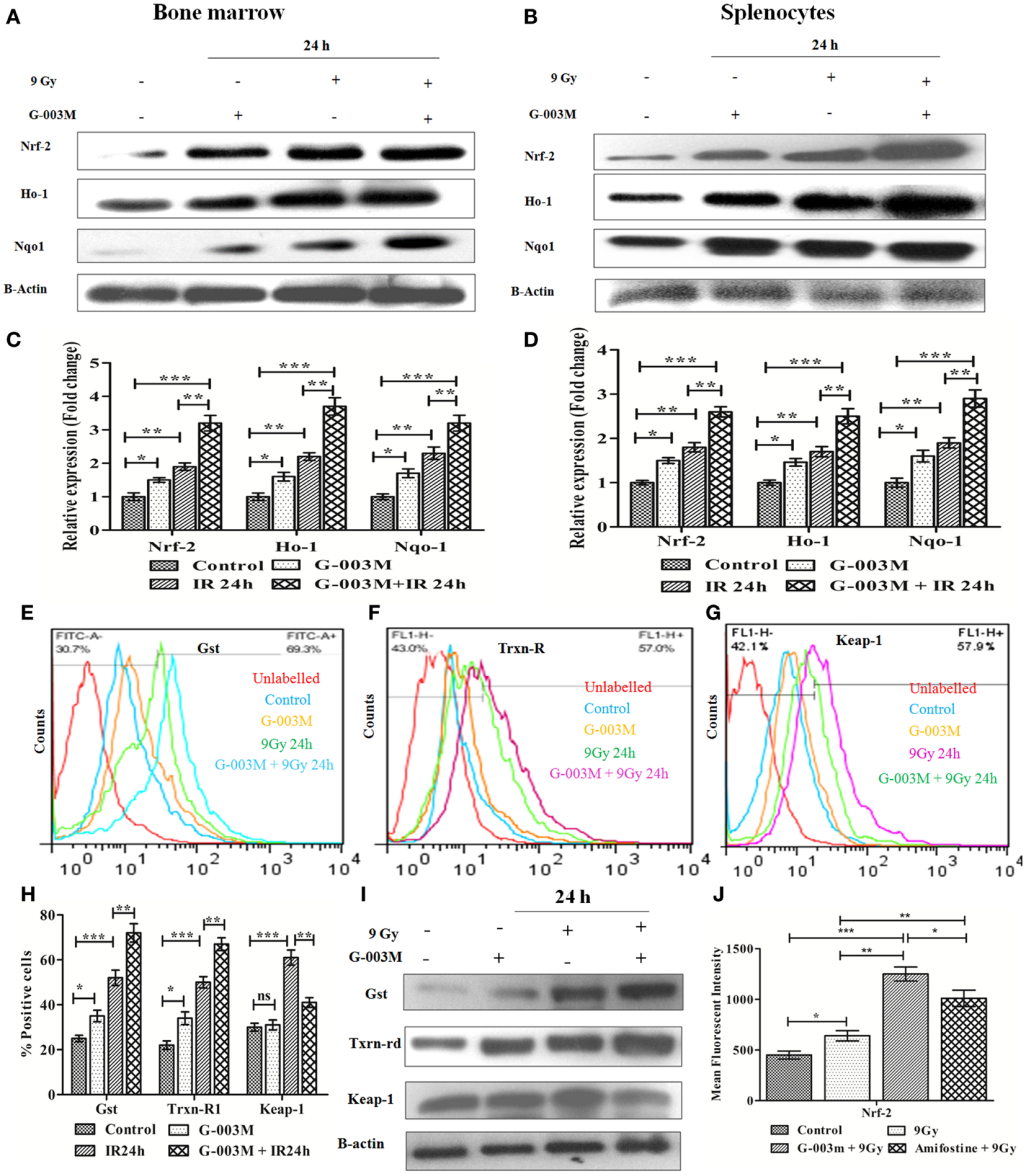
**G-003M preadministration promoted activation of antioxidant pathway in radiation-exposed mice**. **(A)** Immunoblot of antioxidant proteins in bone marrow. **(B)** Immunoblot of antioxidant proteins in splenocytes. **(C)** Densitometry of nuclear factor erythroid-derived like-2 factor (Nrf-2), Heme oxygenase-1 (Ho-1), and Nqo-1 in bone marrow cells. **(D)** Densitometry of Nrf-2, Ho-1, and Nqo-1 in splenocytes. **(E,F,G)** Flow cytometric overlaid histogram demonstrating glutathione *S*-transferase (Gst), thioredoxin reductase-1 (Txnrd-1), and keap-1, respectively, in mice bone marrow. **(H)** Data showing mean fluorescence intensity of proteins in bone marrow. **(I)** Immunoblot of Gst, Txnrd-1, and keap-1 in mice spleen. **(J)** Data depicting level of Nrf-2 in mice bone marrow with use of amifostine as a positive control. Data represent mean ± SEM of six replicate and experiment was repeated twice. Statistical differences among various experimental groups were compared. A value of *P* ≤ 0.5 is considered statistically significant (ns, non-significant, *P ≤ 0.05, **P ≤ 0.01, and ***P ≤ 0.001).

Ionizing radiation-induced oxidative stress subsequently leads to apoptotic cell death. Various studies have demonstrated the cytoprotective potential of Nrf-2 against ROS-induced oxidative stress and subsequent apoptotic cell death. Therefore, we next evaluated whether G-003M has any influences on modulation of these signaling *via* regulation of Nrf-2. For this, we first performed the time kinetic study of various cytoprotective proteins in mice bone marrow and spleen through immunoblotting (Figure S3 in Supplementary Material). Immunoblot study revealed upregulation of Nrf-2 in the bone marrow (Figures 6A,C) and splenocytes of irradiated mice at 24 h post-exposure (Figures 6B,D). However, expression of Nrf-2 got further significantly increased in G-003M pretreated and irradiated group when compared to radiation only group (*P* ≤ 0.01). A similar trend was also obtained in case of Ho-1 and NAD(P)H: quinone oxidoreductase 1 (Nqo1) in both the cellular compartment.

Since, 9 Gy was a lethal dose and delayed study could not be performed due to non-availability of sufficient number of viable cells to extract the proteins. Hence, to distinguish the G-003M and radiation-mediated increase in Nr-2 and Ho-1, we performed delayed expression study (48 h) of both the proteins in bone marrow (Figure S2C in Supplementary Material) and splenocytes (Figure S2D in Supplementary Material) through flow cytometry. The increased value of Nrf-2 and Ho-1 obtained at 24 h post-irradiation substantially declined at 48 h. In G-003M pretreated group, also similar findings were obtained. However, the level of both the proteins was significantly higher in G-003M pretreated group when compared to radiation-only group at 48 h. G-003M-alone treated group also revealed induced level of these proteins when compared to untreated controls. This finding demonstrates the protective potential of our formulation against lethal dose of irradiation at delayed time intervals where antioxidant machinery got paralyzed.

To elucidate whether G-003M pretreatment have any impact on Nrf-2-mediated induction in Gst and Txnrd-1, we have estimated their expression in bone marrow cells (flow cytometry) and splenocytes (immunoblotting). Flow cytometric analysis revealed radiation-mediated increase in Gst and Txnrd-1 at 24 h post-irradiation (*P* ≤ 0.001) (Figures 6E,F,H). G-003M pretreatment further significantly induced Gst and Txnrd-1 level when compared to radiation-exposed group (*P* ≤ 0.01). A similar observation was also obtained while estimating their levels in splenocytes by immunoblotting (Figure 6I). To explore the mechanism of G-003M mediated induction in Nrf-2, expression of keap-1 (Negative regulator of Nrf-2) was also assessed in bone marrow (Figure 6G) and splenocytes (Figure 6I) through flow cytometry and immunoblotting respectively. Keap-1 was significantly increased in both the compartment of irradiated mice over sham group (*P* ≤ 0.001). Pre-irradiation administration of mice with G-003M significantly reduced keap-1 level (*P* ≤ 0.01) in both the bone marrow and spleen. G-003M-alone treated mice also revealed significant downregulation in Keap-1. G-003M thus assisted in amelioration of oxidative stress *via* downregulation of keap-1 and upregulation of Nrf-2 in highly radiosensitive mice bone marrow and spleen.

Nuclear factor erythroid-derived like-2 factor level was also evaluated in bone marrow cells of amifostine pretreated mice. Expression of Nrf-2 in G-003M pretreated group was significantly higher when compared with amifostine pretreated group (*P* ≤ 0.05) (Figure 6J). Nrf-2 level also significantly increased in combination pretreated group when compared with either podophyllotoxin or rutin alone pretreated group. This demonstrates synergy in their mode of action when combined (Figure S4B in Supplementary Material).

### G-003M Spared CD3, CD19, and Gr-1 Cell Surface Receptors

**Figure 7:**
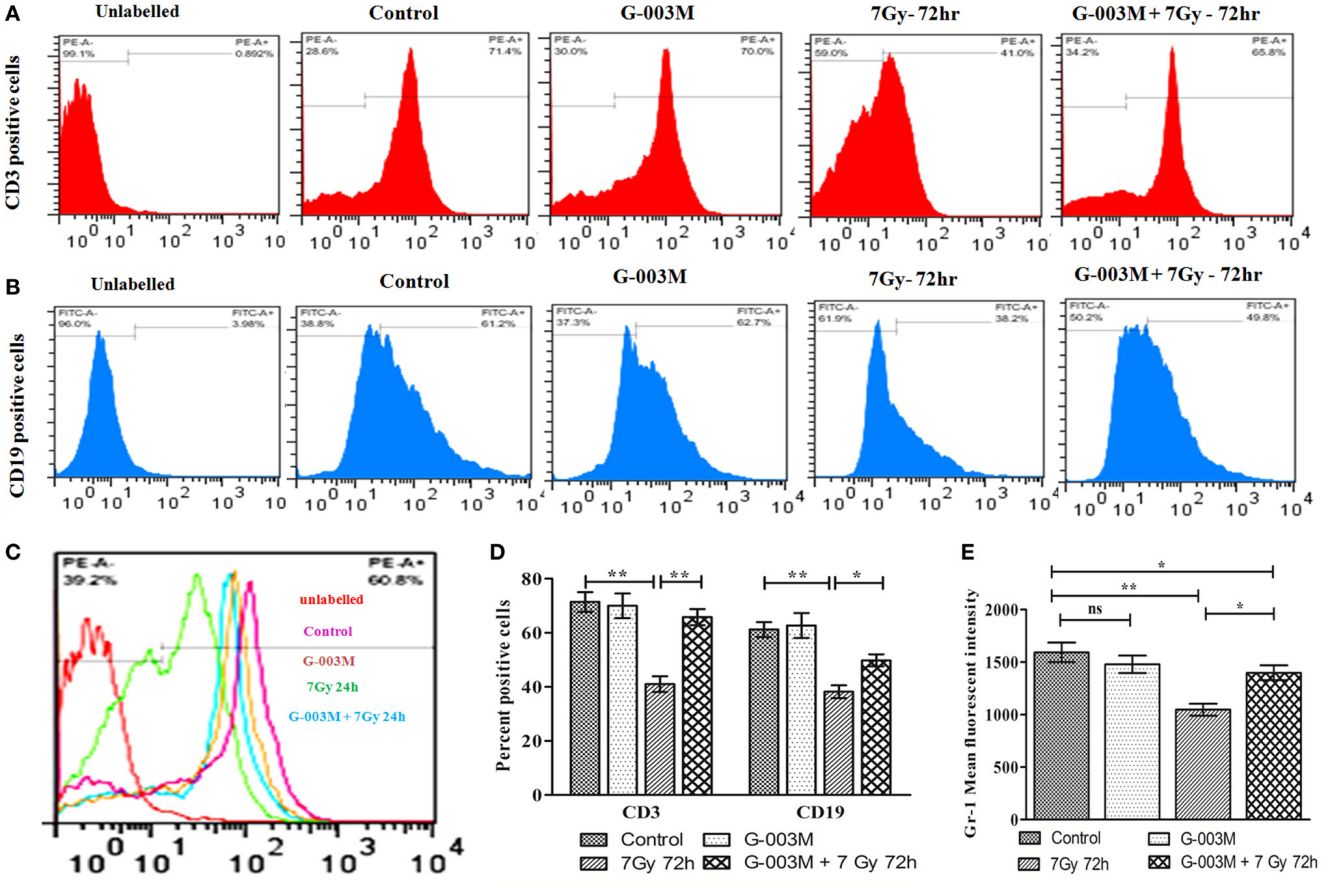
**Modulatory effect of G-003M on level of CD3, CD19, and Gr-1 cell surface marker in splenocytes of irradiated mice**. **(A)** Flow cytometric histogram of CD3 cell surface marker. **(B)** Flow cytometric histogram representing CD19 marker. **(C)** Data showing percent positive cells of CD3 and CD19. **(D)** Flow cytometric histogram revealing Gr-1 level. **(E)** Bar diagram representing Gr-1 mean fluorescent intensity. Data represent mean ± SEM of six mice and experiment was repeated twice. Statistical differences among various experimental groups were compared. A value of *P* ≤ 0.5 is considered statistically significant (ns, non-significant, *P ≤ 0.05, and **P ≤ 0.01).

**Figure 8:**
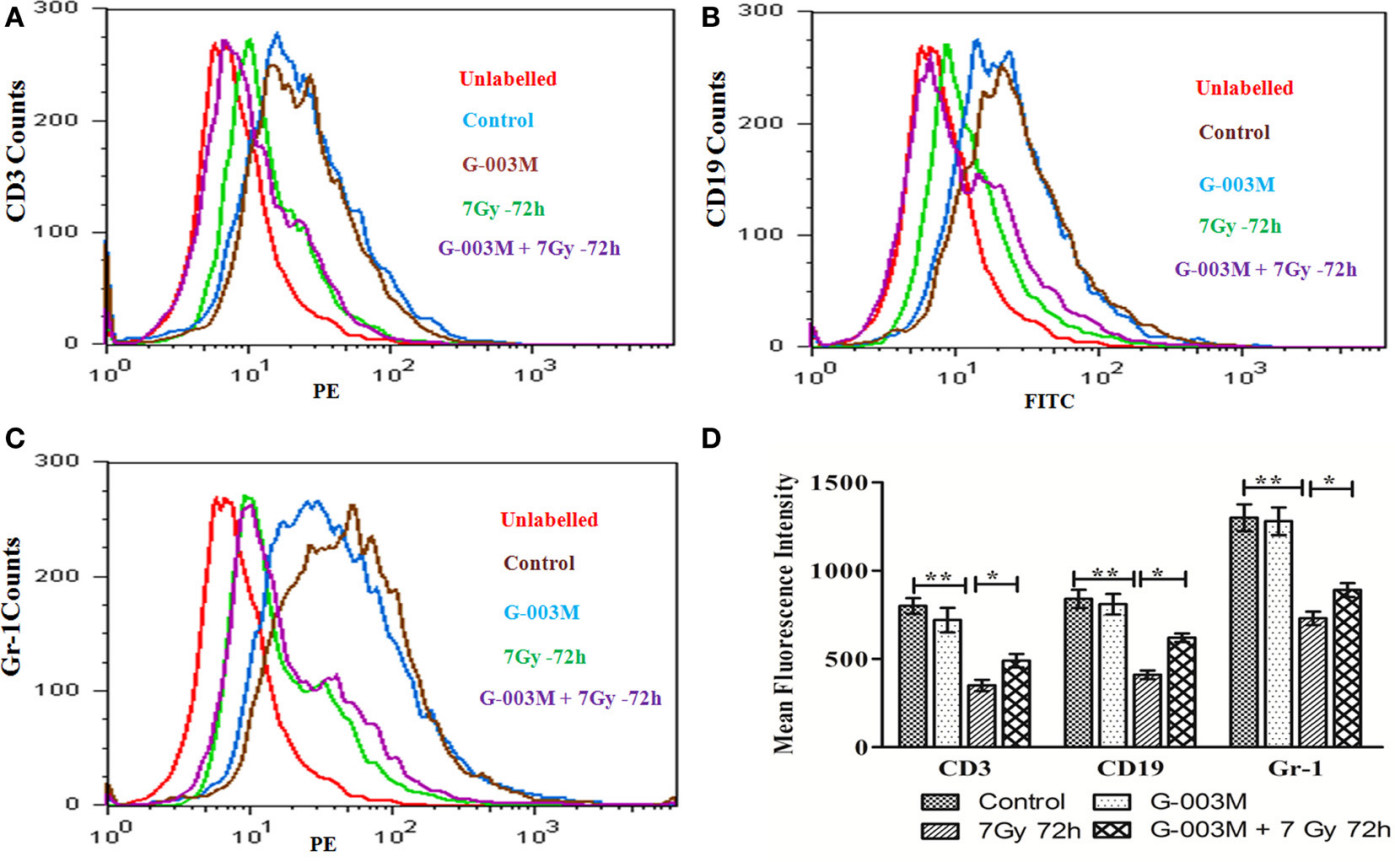
**G-003M-mediated recovery of CD3, CD19, and Gr-1 cell surface receptor in mice bone marrow cells**. **(A)** Histogram showing CD3 level. **(B)** Flow cytometric histogram demonstrating level of CD19. **(C)** Histogram of flow cytometric analysis representing Gr-1 expression. **(D)** Data demonstrating change in mean fluorescent intensity of CD3, CD19, and Gr-1 cell surface markers. Data represent mean ± SEM of six mice and experiment was repeated twice. Statistical differences between various groups were compared. A value of *P* ≤ 0.5 is considered statistically significant (ns, non-significant, *P ≤ 0.05, and **P ≤ 0.01).

To evaluate G-003M-mediated lymphohemopoietic recovery, we have estimated level of CD3, CD19 cells in splenocytes (Figures 7A,B,D), and bone marrow (Figures 8A,B) of differently treated mice. Besides, the expression of Gr-1, a marker of the granulocyte and monocyte was also analyzed in spleen (Figures 7C,E) and bone marrow (Figures 8C,D) of these mice. In mice exposed to 7 Gy, level of CD3 and CD19 was significantly reduced at 72 h post-exposure when compared with control group (*P* ≤ 0.05) in both the organs. G-003M pretreatment to mice provided significant recovery to both the cell lineages over radiation alone group (*P* ≤ 0.05). Further, G-003M also significantly spared Gr-1 in splenocyte and bone marrow compartment when compared to radiation exposed group. Effect of both the compounds, i.e., podophyllotoxin and rutin, individually as well as in combination (G-003M) has also been demonstrated while evaluating the Gr-1 level in mice bone marrow cells (Figure 4C in Supplementary Material). Gr-1 expression was found significantly retained in G-003M pretreated mice when compared with mice either treated with podophyllotoxin or rutin.

## Discussion

Ionizing radiation-inflicted cellular injuries are primarily attributed to deleterious effect of free radicals on cellular DNA, proteins, and lipids (32). To mitigate this, various strategies, including exogenous administration of synthetic compounds, vitamins, antioxidants, and phytocompounds, have been employed over a period of two decades. Many of these synthetic compounds have advanced up to various phases, but till date there is not even a single safe and potential compound in this category. A battery of limitations associated with them has limited their bedside use. Advantage with use of natural compound is associated with negligible side effects by its constituents. In the recent past, a large number of herbs, due to their multivariate properties and synergy in action, have been extensively exploited for their radioprotective ability. Among various plants studied for their radioprotective potential, *P. hexandrum*, inhabitant of the high altitude region of Leh and Laddakh, India, has been studied intricately by our group. Large numbers of formulations prepared out by its rhizomes were explored in *in vitro, in vivo, and ex vivo* model systems against lethal IR. The current study, however, has been exclusively designed to explore the protective potential of our most recent formulation (G-003M). The protective effect of G-003M was evaluated in highly radiosensitive bone marrow and spleen of mice exposed to whole-body irradiation.

G-003M preadministration to mice extended more than 85% survival against a lethal dose of radiation. G-003M revealed a DRF of 1.26, which is considered to be significantly efficacious. Reduced GSH, an intracellular antioxidant molecule from non-protein thiol groups, is known to be involved in direct detoxification of IR-induced radiolytic products. Our study has demonstrated significantly improved GSH level in spleen of whole-body irradiated mice pretreated with G-003M. This finding of ours is in congruence with earlier reports of Mittal et al. (33) and Han et al. (34), where plant and synthetic preparation had improved/retained GSH levels following irradiation. IR-induced membrane lipid peroxidation was also significantly curbed by G-003M pretreatment to mice. This finding has also shown accordance with report of Feinendegen (35).

The current study has also depicted IR-induced ROS generation in bone marrow cells and splenocytes. However, this got significantly curbed with G-003M pretreatment. This study is in correspondence to earlier published report demonstrating efficient scavenging of ROS by melatonin (36). Efficient elimination and detoxification of ROS has taken care of especially by rutin, an important constituent of G-003M. This bioactive compound has also been shown in our earlier studies for scavenging ROS generation by 40–45% in jejunum of lethally irradiated mice (37) and human peripheral blood lymphocytes (38). ROS depletion, reduced malonaldehyde (MDA) formation, and retained GSH level in mice collectively reveals its great antioxidant potential. Revelatory antioxidant property of our formulation has certainly assisted in regulation of radiation-inflicted damage to lymphohemopoietic system.

Ionizing radiation destabilizes MMP (39), which leads to permeabilization of mitochondrial outer membrane through activation of the tumor suppressor protein p53 and members of Bcl-2 family proteins (40). Mitochondrial membrane permeabilization facilitates release of various proapoptotic proteins from mitochondria to cytosol. These proteins in turn activate downstream caspases signaling to execute the IR-induced apoptotic signal. A study by Baek et al. (41) has demonstrated that KR22332 has minimized radiation-induced (8 Gy) apoptosis by retaining the mitochondrial transmembrane potential in HaCat cells. In line with this report, G-003M pretreatment also regulated IR-induced alteration in mitochondrial membrane integrity by maintaining optimum level of MMP in both the bone marrow cells and splenocytes.

8-Hydroxy 2-deoxyguanosine is the marker of direct oxidative damage to DNA. Intensity of DNA damage is directly proportional to the level of 8-hydroxy 2-deoxyguanosine, which in turn corresponds to dose of radiation received. During our study, we observed a significant increase in 8-OH-dG in plasma of irradiated (9 Gy) mice. However, plasma level of 8-OHdG was found significantly reduced in G-003M pretreated mice. This reveals DNA protective ability of G-003M against radiation-induced lethality to lymphohemopoietic sysem. A study by Kawakatsu et al. (42) has also demonstrated reduced 8-OH-dG in urine of nicaraven treated mice following irradiation. G-003M also maintained the viability of bone marrow cells and splenocytes by regulating the number of cells undergoing apoptotic cell death (Annexin V FITC^+^ and PI^−^) following radiation exposure.

G-003M significantly ameliorated the IR-induced qualitative and quantitative loss of hemopoietic stem cells of bone marrow and lymphocytes and granulocytes of spleen by down-regulating the expression of various proapoptotic proteins (p53, Bax, Bak, Puma, and caspases). In addition, this formulation significantly retained level of antiapoptotic protein and also induced Bcl-2/Bax ratio. Regulation of this ratio is known to play key role in decision of cells whether to undergo apoptosis or not. This study is in consonance to earlier reports on exogenous agent-mediated modulation in cell death (43, 44). During this study, p53 level was obtained to be similar in G-003M and amifostine pretreated mice. Antiapoptotic attribute of the formulation is due to reversible cell cycle arrest property (G2/M) of podophyllotoxin present in G-003M (25). DNA damage and faulty repair, the prerequisite of radiation-induced apoptosis and necrosis, was found amended by G-003M pretreatment. Besides, the frequency of initial damage to DNA was prevented by antioxidant potential of rutin present in G-003M.

Oxidative stress has been implicated in a number of pathological disorders and to prevent this activation of antioxidant pathway is considered to be an important event (45). During this process, a wide array of oxidative toxicants is detoxified before they could inflict critical damage to cellular macromolecules (46). Administration of G-003M significantly boosted the cellular antioxidant defense by activating the Nrf-2 protein. Nrf-2 is a member of the Cap and Collar subfamily of b-ZIP transcriptional factor (47). Under stress free condition, Nrf-2 remains bound to actin anchored protein Keap-1 in cytoplasmic compartment. Therefore, keap-1 serves to negatively regulate the expression of Nrf-2 by promoting its degradation by cullin-3 based ubiquitin ligase under basal conditions (48). However, under oxidative stress conditions, Nrf-2 escapes keap-1-mediated proteosomal degradation and translocates to the nucleus (49). In the nucleus, Nrf-2 binds with the antioxidant responsive element (ARE) present in the promoter region of antioxidants (Ho-1, Txnrd-1), detoxificants (Nqo1, Gst), and cytoprotective genes (Bcl-2) and facilitates their transcription (50).

Importance and clinical significance of Nrf-2 in combating the oxidative insult has been revealed by various studies using Nrf-2 knockout mice (51). Our study has also demonstrated increased Nrf-2 expression in bone marrow cells and splenocytes of lethally irradiated mice. Pretreatment of G-003M to mice either alone or in combination with radiation reduced expression of keap-1. Therefore, G-003M offered significant protection to hemotopoietic and immune system by up-regulating cellular antioxidant machinery through Nrf-2. This study is in line with a report on exogenous agent induced Nrf-2 activation (52). Comparative study of G-003M and amifostine in modulating the antioxidant machinery (Nrf-2) has also validated its potent antioxidant property. Nrf-2 level was significantly higher in G-003M pretreated mice in comparison to the amifostine treated group.

Heme oxygenase-1 is known to be involved in the degradation of heme to produce carbon monoxide and bilirubin (53). Due to its role in removing the potent pro-oxidant heme and generating endogenous antioxidant CO and bilirubin, it possesses antioxidant capacity. Nqo-1 provides cellular protection against oxidative stress induced biological complications (54). Nqo-1 also possess ARE sequence in its promoter region and is also known to be regulated by Nrf-2 (55). Our study has revealed G-003M mediated induction in various downstream targets of Nrf-2 (Ho-1 and Nqo-1) in lymphohemopoietic system of mice. In addition, various other reports have also validated our observation by demonstrating increase in antioxidant and phase II detoxifying proteins in different cellular and animal model systems by treatment with different phytomolecules (56, 57).

Thioredoxin reductase-1, the other downstream target of Nrf-2, maintains intracellular redox homeostasis for proper functioning of cellular metabolism and reductive biosynthesis of macromolecules. Gst is also an effector protein of Nrf-2, which is involved in detoxification and elimination of various toxicants by conjugation reaction with GSH. This makes toxicants more hydrophilic and thus facilitates their removal (58). In the current study, we have demonstrated that G-003M pretreatment facilitated significant increase in expression of these proteins. Our study is in congruence to a report by Patil et al. (59) showing phytocompound mediated induction of Gst and Txnrd-1.

Ionizing radiation-induced lymphoid and hematopoietic injury is the major cause of post-irradiation mortality (8). Survival of the animal depends majorly on the availability of remaining viable hematopoietic stem cells after irradiation and their potential to replenish. CD19 gene encodes a cell surface molecule that assembles with the low affinity antigen receptor of B-lymphocytes. CD19 receptor is present on membrane of B cells throughout its developmental stages and serves as a marker of B-lymphocytes. CD3 is the T-cell co-receptor and helps in activation of cytotoxic-T cells. However, CD3 unlike CD19 is only present on membrane of all mature T-cells and act as their marker. Gr-1, a cell surface receptor, is present on granulocytes and monocytes. Radiation exposure lead to significant reduction in expression of these cell surface receptors (CD3, CD19, and Gr-1) in bone marrow and splenocytic compartment due to severe damage to different lineages of precursor cells residing in the bone marrow. G-003M pretreatment, however, extended significant recovery to bone marrow and spleen cell by retaining expression of these cell surface receptors. G-003M thus assisted in protection and recovery to stem cells of bone marrow, which has subsequently led to the maintenance of myeloid and lymphoid lineages in peripheral depot, suggesting its immune reconstituting properties.

In the present study, we elucidated that G-003M has a potential to replenish IR-induced damage in bone marrow and splenocytes *via* induction of cellular antioxidant machinery. G-003M-mediated strengthening of radioresistance of hemopoietic stem and immune cells through the modulation of p53-dependent cell death pathway was also revealed. In addition, G-003M extended significant protection and recovery to bone marrow and spleen by retaining the level of CD3, CD19, and Gr-1 cell surface receptors against radiation-induced immunosuppression. Observation of the current study suggests that G-003M administration can prolong survival of lethally exposed mice by enhancing the regeneration of hemopoietic stem cells in bone marrow and also by promoting immune function of splenic lymphocytes. This could predominantly occur by G-003M-mediated modulation of IR-induced oxidative stress and cell death pathways in bone marrow and spleen.

## Author Contributions

Conceived, designed, and performed the experiments: AS and MG. Analyzed the data: MY, BK, and RR. Contributed reagents/materials/analysis tool: MY, BK, and SB. Wrote the paper: MG and AS. Manuscript editing: HP.

## Conflict of Interest Statement

The authors report no declaration of interest. The authors are solely responsible for the content and writing of the paper.

## Supplementary Material

Figure S1 **Modulation of ionizing radiation (IR)-induced reactive oxygen species (ROS) generation and alteration in mitochondrial membrane potential (MMP) by G-003M administration**. **(A)** Flow cytometric overlaid histogram depicting ROS level in bone marrow. **(B)** Flow cytometric overlaid histogram showing level of ROS in spleen. **(C)** Flow cytometric overlaid histrogram demonstrating MMP level in bone marrow. **(D)** Flow cytometric histogram representing MMP level in spleen.

Figure S2 **Estimation of ionizing radiation (IR)-induced apoptotic cell death and measurement of nuclear factor erythroid-derived like-2 factor (Nrf-2) and heme oxygenase-1 (Ho-1) in different experimental groups**. 1 × 10^−6^ viable cells were stained with annexin V/PI. The percentage of apoptotic cells was measured by flow cytometry. Apoptotic cells (Annexin V^+^ and PI^−^) are displayed in upper left quadrant and necrotic cells (Annexin V^+^ and PI^+^) are shown in upper right quadrant. **(A)** Flow cytometric dot plot in bone marrow cells. **(B)** Flow cytometric dot plot in splenocytes. **(C)** Data showing level of Nrf-2 and Ho-1 in mice bone marrow at 24 and 48 h post-exposure. **(D)** Level of Nrf-2 and Ho-1 in mice spleen at 24 and 48 h post-irradiation.

Figure S3 **Effect of whole-body irradiation (9 Gy) on expression levels of various proapoptotic and cytoprotective proteins at various time intervals (6, 12 and 24 h)**. **(A)** Immunoblot-based time kinetics in bone marrow cells. **(B)** Bar diagram represents the densitometry of proapoptotic proteins in bone marrow cells. **(C)** Bar diagram showing the densitometry of cytoprotective proteins in bone marrow cells. **(D)** Time kinetics study by immunoblotting in splenocytes. **(E)** Densitometry of proapoptotic proteins in splenocytes. **(F)** Bar diagram showing expression level of cytoprotective proteins in splenocytes. Data showing mean ± SEM of six replicates and experiment was repeated twice.

Figure S4 **Individual effect of podophyllotoxin, rutin, and their combination (G-003M) on level of p53, nuclear factor erythroid-derived like-2 factor (Nrf-2), and Gr-1 in bone marrow cell of irradiated mice**. **(A)** Flow cytometric histogram of p53 in bone marrow. **(B)** Histogram of Nrf-2 in bone marrow. **(C)** Flow cytometric overlaid histogram of Gr-1 in mice bone marrow. Panel **(D)** demonstrates number of animal used in different experimental groups of various study parameter.
