# Supplementary figures and images for: Podophyllotoxin and Rutin Modulates Ionizing Radiation-Induced Oxidative Stress and Apoptotic Cell Death in Mice Bone Marrow and Spleen
Source: Front Immunol. 2017 Feb 27;8:183. doi: 10.3389/fimmu.2017.00183 (PMC5326804; doi:10.3389/fimmu.2017.00183)

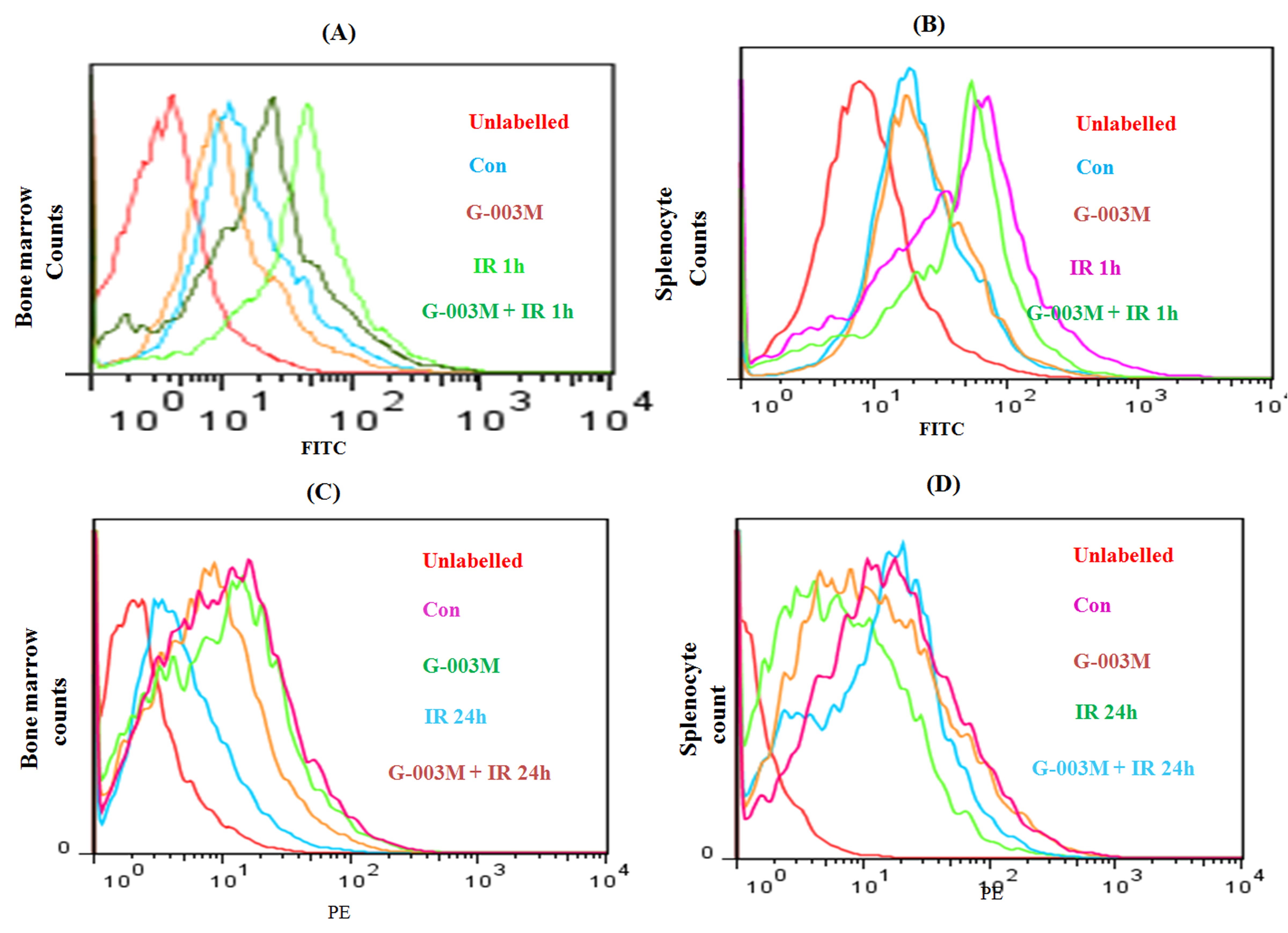

Supplement: Figure S1 — Modulation of ionizing radiation (IR)-induced reactive oxygen species (ROS) generation and alteration in mitochondrial membrane potential (MMP) by G-003M administration. (A) Flow cytometric overlaid histogram depicting ROS level in bone marrow. (B) Flow cytometric overlaid histogram showing level of ROS in spleen. (C) Flow cytometric overlaid histrogram demonstrating MMP level in bone marrow. (D) Flow cytometric histogram representing MMP level in spleen. [file Image_1.TIF]

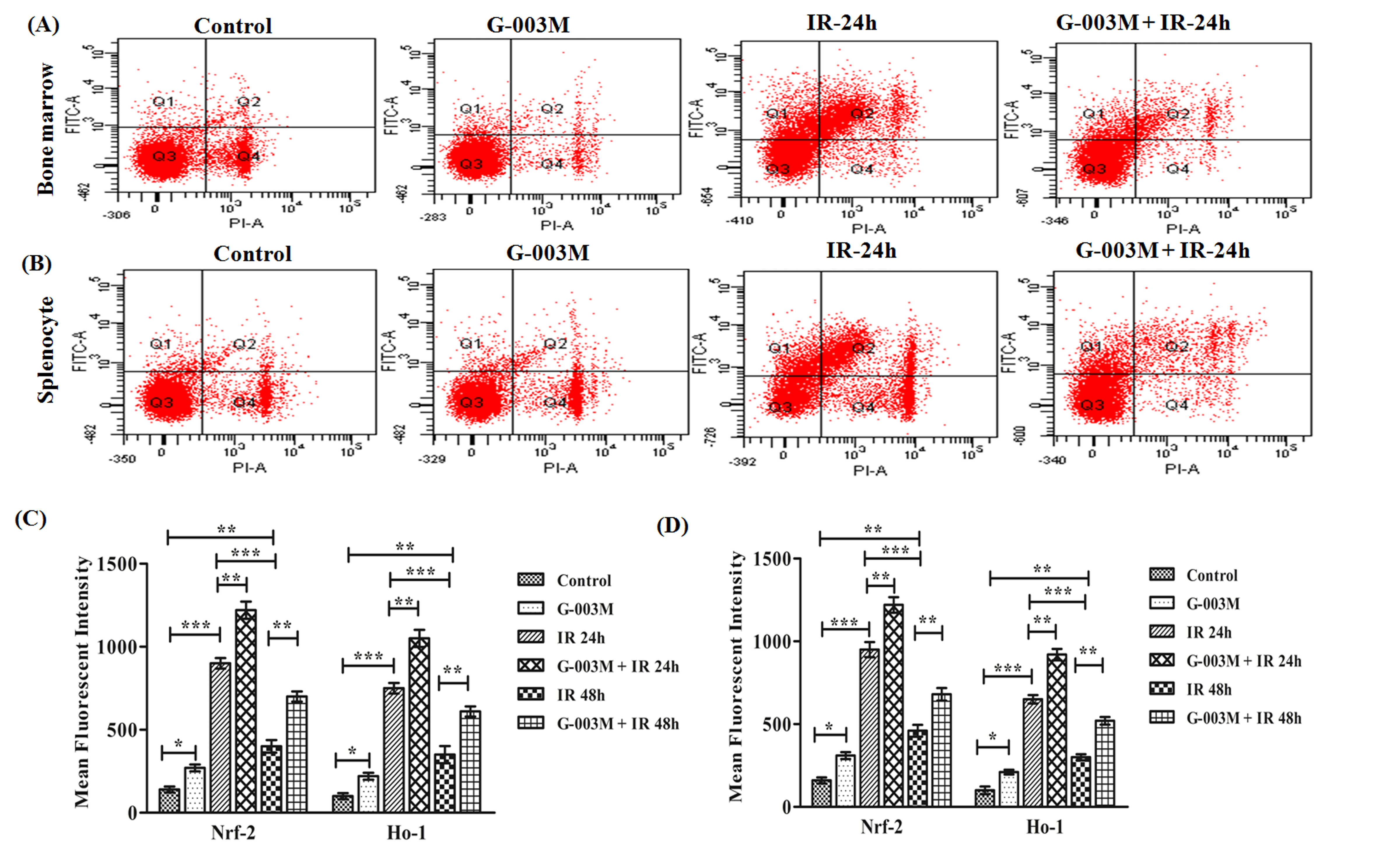

Supplement: Figure S2 — Estimation of ionizing radiation (IR)-induced apoptotic cell death and measurement of nuclear factor erythroid-derived like-2 factor (Nrf-2) and heme oxygenase-1 (Ho-1) in different experimental groups. 1 × 10^−6^ viable cells were stained with annexin V/PI. The percentage of apoptotic cells was measured by flow cytometry. Apoptotic cells (Annexin V+ and PI−) are displayed in upper left quadrant and necrotic cells (Annexin V+ and PI+) are shown in upper right quadrant. (A) Flow cytometric dot plot in bone marrow cells. (B) Flow cytometric dot plot in splenocytes. (C) Data showing level of Nrf-2 and Ho-1 in mice bone marrow at 24 and 48 h post-exposure. (D) Level of Nrf-2 and Ho-1 in mice spleen at 24 and 48 h post-irradiation. [file Image_2.TIF]

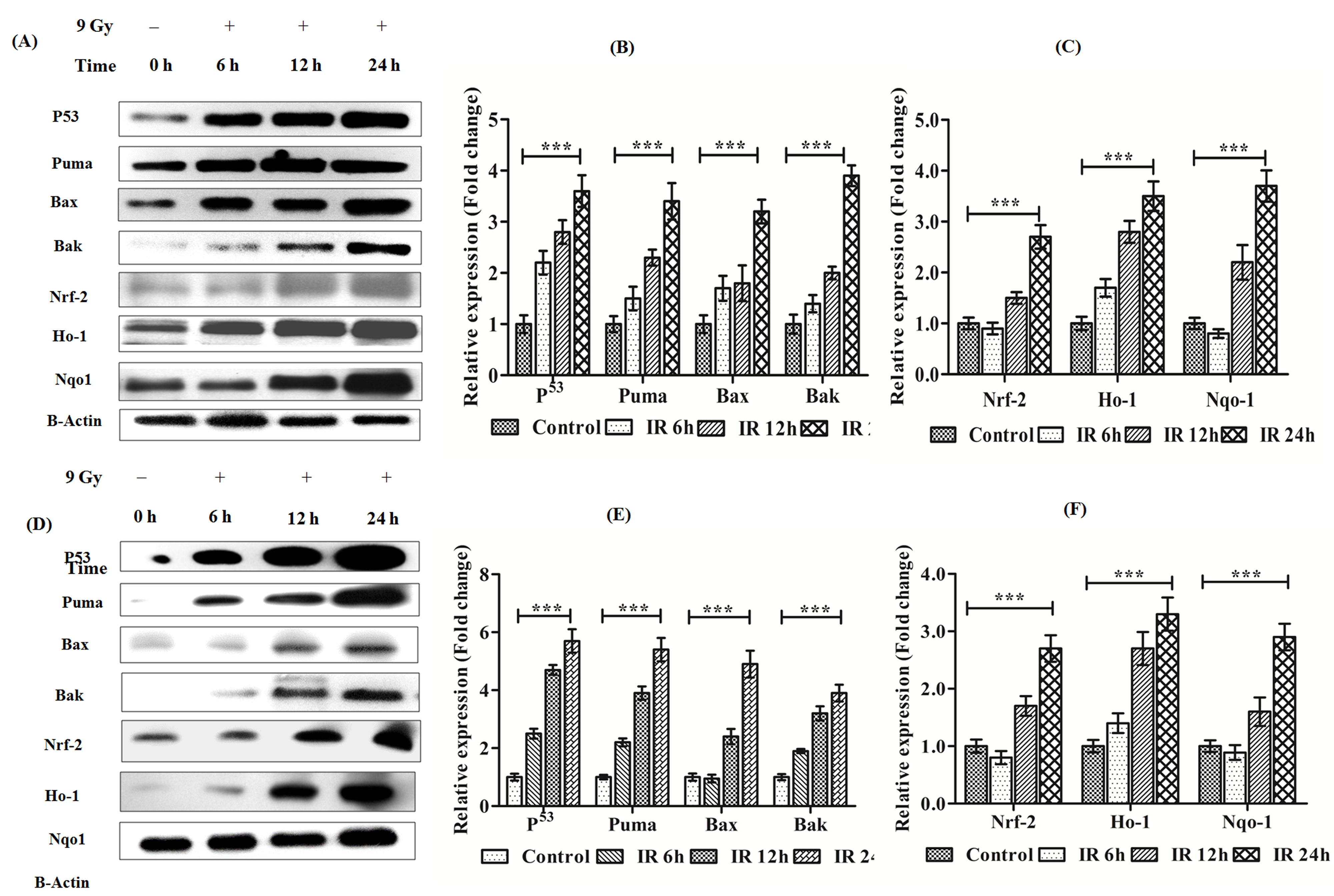

Supplement: Figure S3 — Effect of whole-body irradiation (9 Gy) on expression levels of various proapoptotic and cytoprotective proteins at various time intervals (6, 12 and 24 h). (A) Immunoblot-based time kinetics in bone marrow cells. (B) Bar diagram represents the densitometry of proapoptotic proteins in bone marrow cells. (C) Bar diagram showing the densitometry of cytoprotective proteins in bone marrow cells. (D) Time kinetics study by immunoblotting in splenocytes. (E) Densitometry of proapoptotic proteins in splenocytes. (F) Bar diagram showing expression level of cytoprotective proteins in splenocytes. Data showing mean ± SEM of six replicates and experiment was repeated twice. [file Image_3.TIF]

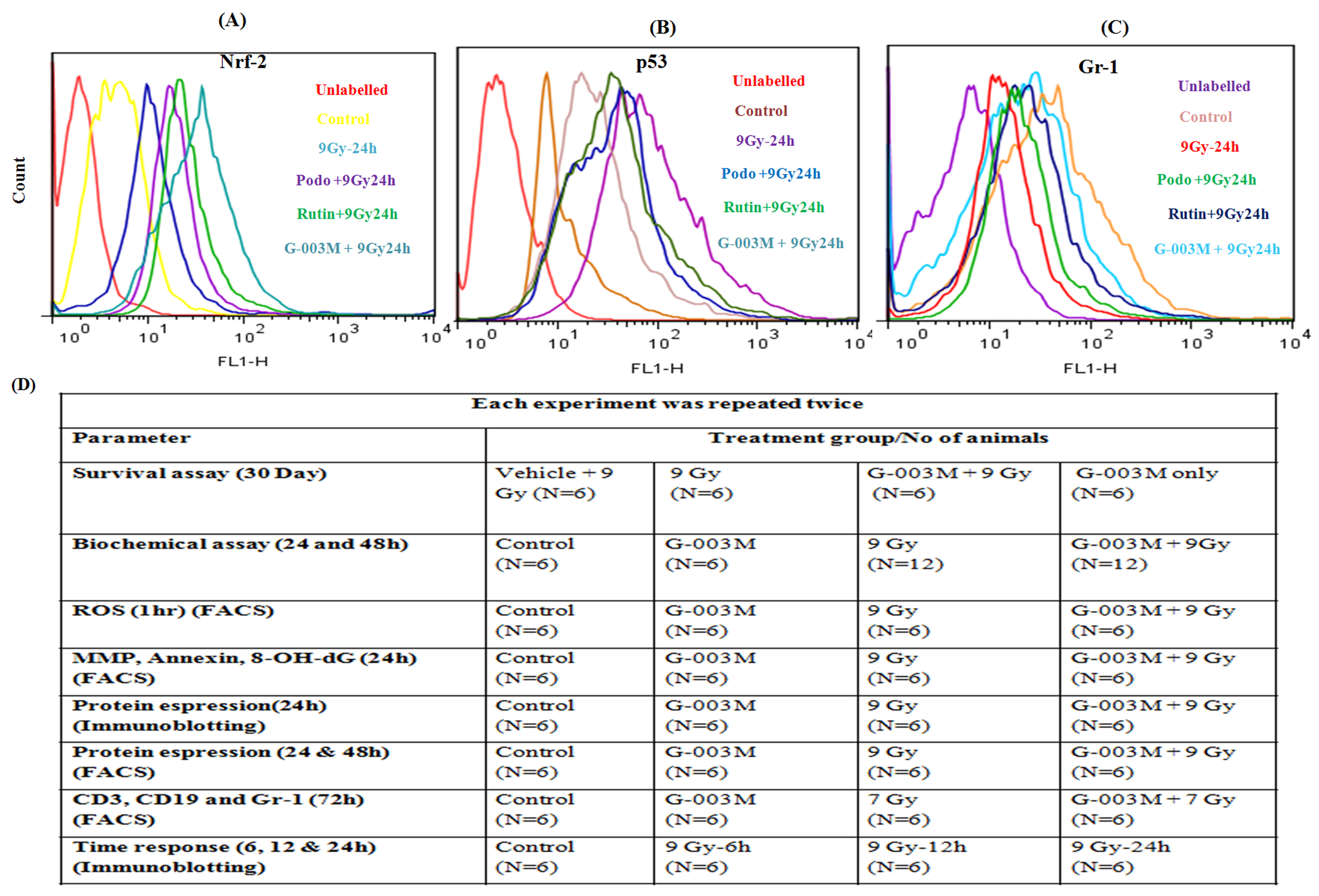

Supplement: Figure S4 — Individual effect of podophyllotoxin, rutin, and their combination (G-003M) on level of p53, nuclear factor erythroid-derived like-2 factor (Nrf-2), and Gr-1 in bone marrow cell of irradiated mice. (A) Flow cytometric histogram of p53 in bone marrow. (B) Histogram of Nrf-2 in bone marrow. (C) Flow cytometric overlaid histogram of Gr-1 in mice bone marrow. Panel (D) demonstrates number of animal used in different experimental groups of various study parameter. [file Image_4.TIF]
